# Timing of First Cuddling After Preterm Birth Improved Over Five Decades, While Delays Were Linked to Negative Maternal Experiences

**DOI:** 10.1111/apa.70304

**Published:** 2025-09-27

**Authors:** Achim Fieß, Alica Hartmann, Alexander K. Schuster, Mareike Ernst, Eva Mildenberger, Dirk Wackernagel, Stephanie D. Grabitz, Michael S. Urschitz, Norbert Pfeiffer, Manfred E. Beutel, Sandra Gißler, Jonas Tesarz

**Affiliations:** ^1^ Department of Ophthalmology University Medical Center of the Johannes Gutenberg University Mainz Mainz Germany; ^2^ Department of Psychosomatic Medicine and Psychotherapy University Medical Center of the Johannes Gutenberg University Mainz Mainz Germany; ^3^ Department of Clinical Psychology, Psychotherapy and Psychoanalysis, Institute of Psychology University of Klagenfurt, Klagenfurt Am Wörthersee Austria; ^4^ Division of Neonatology, Department of Pediatrics University Medical Center of the Johannes Gutenberg‐University Mainz Mainz Germany; ^5^ Division of Paediatric Epidemiology, Institute of Medical Biostatistics, Epidemiology, and Informatics University Medical Centre of the Johannes Gutenberg University Mainz Mainz Germany; ^6^ Department of General Internal Medicine and Psychosomatics University Hospital Heidelberg, Heidelberg University Heidelberg Germany; ^7^ DZPG (German Centre for Mental Health – Partner Site Heidelberg/Mannheim/Ulm) Germany

**Keywords:** maternal psychological birth trauma, neonatal intensive care, parental discharge preparedness, preterm birth, timing of skin‐to‐skin contact

## Abstract

**Aim:**

Early physical contact after birth is critical for families of preterm infants, who face higher risks of psychological stress. This study examined changes in first cuddling over five decades and its relation to parental birth experiences and perceptions of early parenthood.

**Methods:**

This retrospective cohort study was conducted at the University Medical Center Mainz, Germany. Parents completed questionnaires and structured interviews about birth experiences, timing of first cuddling and perceptions of parenthood. Multivariable logistic regression was used to analyse associations of gestational age and cuddling delay with parental outcomes.

**Results:**

Data were obtained from 940 mothers and 614 fathers of 1559 children (median age 15 years, range 4–52, 824 female). Time to first cuddling in extremely preterm infants decreased from several weeks in the 1970s to about 1 week in the 2010s. Mothers of preterm or delayed‐cuddling infants more often perceived birth as an emergency, felt less prepared for discharge and reported negative feelings towards motherhood. Fathers showed similar but weaker associations.

**Conclusion:**

The time until first cuddling decreased markedly over five decades, reflecting greater clinical awareness. However, delayed cuddling remained strongly linked to negative maternal perceptions, underlining the importance of prioritising early physical contact in neonatal care.

**Abbreviations**
AGA: appropriate for gestational age
GPS: Gutenberg Prematurity Study
GPSY: Gutenberg Prematurity Study Young
LGA: large for gestational age
SGA: small for gestational age

**Summary**

- Preterm birth increases parental stress. Cross‐decade evidence on the timing of first cuddling and links to parental experiences has been limited.
- In 1559 births, time to first cuddling shortened across decades, reaching about 1 week in the 2010s for extremely preterm infants and delays were linked to negative maternal experiences.
- Prioritising early cuddling after preterm birth may improve parental experience and discharge preparedness, and future research should evaluate implementation.

## 1 Introduction

Children born preterm are at risk of developing depression [1], anxiety disorders [2] and ongoing mental health problems in adulthood including panic disorder [3]. At the same time, preterm birth can have a profound psychological impact on parents. It is often experienced as traumatic because of its sudden onset and the intensive medical environment [4, 5, 6]. The resulting early separation between infant and parents, frequently due to neonatal intensive care, can strain the bonding process. Bonding is essential for the healthy emotional and neurological development of newborn infants [7, 8, 9]. It is generally understood as the early emotional connection from parent to infant, typically forming shortly after birth. In contrast, attachment develops later and is shaped by the infant's experiences with caregivers over time [10]. Early physical closeness between parents and newborns plays a critical role in facilitating bonding, reducing parental stress and supporting infant stability. Two concepts have emerged in neonatal care to promote this early closeness: skin‐to‐skin contact and Kangaroo care. According to the World Health Organization [11], skin‐to‐skin contact refers specifically to the practice of placing the naked newborn on the bare chest of a parent to encourage warmth, regulation and bonding. Kangaroo care describes a broader approach that includes continuous skin‐to‐skin contact, exclusive breastfeeding, early discharge from hospital and follow‐up support.

In this study, we use the term cuddling to refer to the first moment of physical closeness between an infant and a parent. It is defined as the first skin‐to‐skin contact after birth, regardless of duration. Unlike Kangaroo Care, which encompasses a package of interventions, and unlike continuous or protocol‐guided skin‐to‐skin routines, cuddling here refers to the initial intentional physical contact after giving birth. This moment may be brief or prolonged and can occur hours, days, or weeks after birth depending on the infant's medical condition.

Delays in cuddling, especially in preterm infants, are common due to medical stabilisation needs. Evidence indicates that postponed initial contact may influence maternal well‐being and the parent–infant relationship [12, 13, 14, 15]. Scandinavian research has shown that immediate skin‐to‐skin contact for very preterm infants reduced symptoms of parental anxiety and depression after birth [16]. Studies of Kangaroo Care also demonstrated that early physical closeness is associated with improved maternal mood, enhanced infant development and better long‐term parent–child interactions [17, 18, 19, 20]. One previous analysis of the Gutenberg Prematurity Study (GPS) found that delayed first cuddling was associated with children recalling their mothers as more rejecting, overprotective and less emotionally warm [21]. Early parent–child interactions were once undervalued. Their importance later gained recognition through intervention studies and advances in developmental psychology, neuroscience and attachment theory [22]. Nevertheless, individuals born preterm and their parents are still underrepresented in such research [23].

The aim of this study was to examine the timing of first cuddling across five decades in individuals born preterm between 1969 and 2018. Specifically, it investigated whether delayed first physical contact was associated with parental birth experiences and long‐term emotional perceptions. The analysis further differentiated between degrees of prematurity and foetal growth restriction in order to capture potential variation in parental birth experiences and long‐term perceptions.

## 2 Methods

### 2.1 Study Population

This analysis was based on retrospective interviews with parents of individuals enrolled in the GPS and the Gutenberg Prematurity Study Young (GPSY) at the University Medical Center Mainz, Germany. The source population comprised persons born preterm or at term at the University Medical Center Mainz between 1969 and 2018. The two studies were cohort studies with prospective examinations. GPS enrolled adults and collected examinations and interviews between 2019 and 2021 [24]. GPSY enrolled children and adolescents. The participants underwent examinations between 2022 and 2023 and provided additional information about their medical history through surveys [25]. After each index participant completed a study visit, we contacted one or both parents. They were invited to complete a retrospective questionnaire and, if desired, a structured interview about the birth and early development. For the present analysis, we focused on these parental reports. Throughout the manuscript, we referred to the sons and daughters enrolled in the two studies as the index cohort and to the reporting mother or father as the parent.

The parents were invited to complete a questionnaire regarding their experiences surrounding the birth of their child, including the timing of first physical contact after giving birth. All reported experiences and perceptions are based on parental self‐report referring to the birth and early postnatal period of the index cohort members. Both mothers and fathers were contacted; however, a higher proportion of mothers agreed to participate. Parents of adult participants (≥ 18 years) were invited via their children, who provided contact information or forwarded the invitation themselves. For participants under the age of 18, parental contact and consent were obtained directly.

The original index cohort was recruited as follows. At the University Medical Center, we invited every second individual born preterm at 33–36 weeks' gestation for the GPS cohort and every third infant for the GPSY cohort between 1969 and 2018. We also invited all individuals born at 32 weeks' gestation or less. For each birth month from 1969 to 2018, we selected six individuals born at term, three male and three female, with a birth weight between the 10th and 90th percentile for the GPS cohort. In addition, we selected eight full‐term infants, four male and four female, for the GPSY cohort. They formed a control group matched for age and sex. To ensure a broad and representative range of birth weight percentiles within the cohort, we additionally invited full‐term individuals born with varying degrees of foetal growth restriction or overgrowth. Individuals born at term were classified by birth weight as small for gestational age (SGA) or large for gestational age (LGA). Categories were severe SGA, below the 3rd percentile; moderate SGA, 3rd to 10th percentile; moderate LGA, 90th to 97th percentile; and severe LGA, above the 97th percentile. In the present study, we used birth weight percentile as a continuous variable to preserve statistical power and to examine the role of foetal growth independent of gestational age. This allowed us to explore whether both gestational age and intrauterine growth deviations were associated with the outcomes of interest. The use of birth weight percentiles is a standard approach in perinatal research to reflect variations in foetal nutrition and growth [26, 27]. Individuals in the index cohort were matched by age (in years) and sex.

The original index cohort was stratified by gestational age into four groups (Figure S1). Group 1 included 680 individuals born at 37 + 0 weeks' gestation or later, and Group 2 included 379 born at 33 + 0 to 36 + 6 weeks' gestation. Group 3 included 296 born at 29 + 0 to 32 + 6 weeks' gestation, and Group 4 included 204 born before 29 + 0 weeks' gestation. Between 2019 and 2023, these individuals underwent comprehensive medical evaluations and took part in interviews. By birth decade, the cohort comprised seven individuals from 1969 to 1970, 86 from the 1970s, 148 from the 1980s, 271 from the 1990s, 562 from the 2000s and 485 from the 2010s.

Parental participation decreased with increasing time since birth. In particular, it was more challenging to reach parents of individuals born in the 1960s and 1970s due to advanced age, health limitations, or loss to follow‐up. For example, parents of individuals born in 1969 would have been approximately 75–85 years old at the time of the study. Despite these challenges, parental interviews were conducted across all decades, although with lower response rates in earlier cohorts. In total, 418 mothers participated in Group 1, 230 in Group 2, 171 in Group 3 and 121 in Group 4. There were 276 fathers in Group 1, 161 in Group 2, 96 in Group 3 and 81 in Group 4. Due to the lower number of fathers, the main analysis focused on mothers.

### 2.2 Data Collection Process

Initially, participants in GPS and GPSY completed a standardised sociodemographic questionnaire and underwent a clinical interview and physical examination. For this analysis, we invited their parents to complete a retrospective questionnaire on the early postnatal period, including the timing of first physical contact. Parents could complete the questionnaire on paper or online at home. We also offered a structured interview by telephone or on site at the study centre to clarify questions and supplement missing information. Parents who required support or had follow‐up questions were invited to attend the study centre in person.

### 2.3 Cuddling Definition

In this study, cuddling was defined as the first instance of skin‐to‐skin contact between the newborn and a parent, regardless of duration or continuity. This definition includes both brief and prolonged physical contact and is based on retrospective parental self‐report, rather than clinical documentation. This retrospective perspective was essential because the opportunity for early physical contact between parents and newborns has evolved significantly over the last five decades. In earlier periods, particularly during the 1970s and 1980s, parental involvement in neonatal units was limited and physical closeness was often delayed or restricted due to medical concerns, lack of protocols, or institutional culture. From the 1990s, and more so in the 2000s, many neonatal units in Germany, including ours, adopted family‐centred care. This allowed earlier and more consistent parent–infant interaction. This transition was often driven by engaged nursing staff and supported by advances in neonatal care and interdisciplinary collaboration. Therefore, we defined cuddling broadly to accommodate this variation. It included any early physical contact as experienced and recalled by parents, regardless of whether it met contemporary clinical definitions of skin‐to‐skin contact.

Parents were asked when they were first allowed to cuddle their child or when the child was placed on their chest. Response options were: day of birth, first week defined as days two to seven, second week defined as days 8–14, third week starting on day 15 or later with the number of weeks specified. We recoded answers to a continuous measure of weeks to first cuddling and used this in all inferential analyses. For descriptive figures, time to first cuddling was grouped as day of birth, week one, week two and after week two.

While bonding and attachment are often discussed in the context of early parent–infant interaction, our study does not aim to measure these processes directly. We refer to bonding as part of the theoretical framework for understanding why the timing of first physical contact may be perceived as emotionally significant by parents. No standardised bonding or attachment instruments were used.

### 2.4 Experiences of Childbirth

Peritraumatic distress was assessed using the Peritraumatic Distress Inventory, a validated self‐report instrument developed by Brunet et al. [28] to measure emotional and physiological distress experienced during or immediately after a potentially traumatic event. In this study, total scores on the Peritraumatic Distress Inventory were dichotomised using a cut‐off score of ≥ 23 to identify clinically significant levels of peritraumatic distress. This threshold was based on the findings of Bunnell et al. [29], who demonstrated that a score of 23 best predicted clinically elevated PTSD symptoms 30 days after trauma exposure, with acceptable sensitivity (71%) and specificity (73%). This binary classification was used in all subsequent analyses to simplify interpretation and focus on clinically meaningful distress. To characterise the birth context and perceived urgency, we asked parents whether they had experienced the circumstances surrounding the birth as an emergency. This item captured the immediate subjective appraisal of the situation. Responses were yes or no.

To assess perceived long‐term effects, parents were asked whether the circumstances at birth had a lasting impact on their child's life during childhood. They were also asked whether their child still experienced ongoing impairments attributable to the birth, including consequences of prematurity and of low or high birth weight. In addition, parents indicated whether they felt sufficiently prepared at the time of hospital discharge after birth. All items used yes or no response options. The items were developed for this study and have not been validated. They were intended to capture parents' subjective perceptions of emotional and developmental consequences related to the birth, including medical complications and early separation.

### 2.5 Perception of Parenthood

Emotional responses to parenthood were assessed by asking parents whether they had frequently experienced negative feelings related to being a parent. Parents indicated whether such feelings had occurred around the time of birth, during their child's childhood, or not at all. This item was intended to capture a broad range of self‐perceived emotional distress related to parenting, particularly in the perinatal period and early child development.

### 2.6 Evaluation of Prenatal and Postnatal History

The medical records of the participants archived at the University Medical Center Mainz contained parameters including gestational age in weeks and birth weight in kilograms. Additional factors were assessed by medical chart reviews and maternal interviews such as placental insufficiency, maternal smoking, preeclampsia and breastfeeding history. Since this study is based on a German cohort, the growth charts by Voigt et al. were chosen as they provide population‐specific reference values for birth weight percentiles in Germany [30]. Data regarding the postnatal clinical course included the time spent in the neonatal intensive care unit in days. Perinatal medical charts were used to record Apgar scores taken at 5 min.

### 2.7 Statistical Analysis

The absolute and relative frequencies were determined for dichotomous parameters, with the mean and standard deviation calculated for normally distributed variables, and the median and interquartile range were determined for non‐normally distributed variables. Analysis of variance (ANOVA) was used to analyse differences in continuous variables across gestational age groups and the chi‐square test was used for categorical variables, calculating global *p* values. The significance level was set at 5%.

Multivariable logistic regression was used to analyse primary outcomes reported by the mothers: birth experience as an emergency, parental psychological birth trauma and discharge preparation from neonatal care. Additional analyses examined the association between birth conditions and outcomes, specifically focusing on impairment in childhood due to birth and negative feelings towards motherhood at birth or during the child's later childhood. The analyses included three models. Model 1 used gestational age categories of 28 weeks or less, 29–32 weeks and 33–36 weeks. Model 2 added birth weight percentile. Model 3 further included Apgar score at 5 min, weeks until first cuddling, and duration of neonatal intensive care unit stay. A gestational age of ≥ 37 weeks was used as the reference category for the gestational age groups. All analyses were adjusted for maternal age and for the child's age at the time of the survey to account for potential effects of time since birth and developmental stage on parental reporting. Older maternal age has been associated with increased obstetric risk [31, 32] and may shape perceptions of the birth experience. Similarly, parental recall of birth‐related experiences may be shaped by the time elapsed since the event, with longer intervals increasing recall bias. Child age was therefore included as a proxy for time elapsed since birth to help account for historical differences in neonatal care. Furthermore, maternal and paternal reports were analysed separately to assess differences in perceptions and outcomes between mothers and fathers. To ensure sufficient power in the analysis due to fewer paternal data, the gestational age deficit (the difference in weeks between the infant's actual gestational age and full term, indicating the degree of prematurity) was used as a continuous parameter instead of gestational age categories.

This is an exploratory study, so no adjustment for multiple testing was performed. All statistical analyses were conducted using R version 4.3.2 (R Foundation, Vienna, Austria).

### 2.8 Ethics

All parental participation was voluntary and based on written informed consent. The retrospective nature of the data collection, particularly the long interval between birth and parental contact, was carefully considered and reviewed by the responsible ethics committee. No concerns were raised regarding the timing of the contact, as all participants were contacted in a respectful and nonobligatory manner. The study complied with all relevant standards of research ethics. All participants provided written informed consent prior to joining the study, adhering to the standards of Good Clinical Practice, Good Epidemiological Practice and the ethical guidelines of the Declaration of Helsinki. All children and parents gave the permission to link their data. The study's protocol and related documents received approval from the local ethics committee of the Medical Chamber of Rhineland‐Palatinate, Germany, under reference no. 2019–14 161 (original approval: 29.05.2019, most recent update: 02.04.2020) and reference no. 2021–15 830 (original approval: 05.05.2021, latest update: 19.01.2022).

## 3 Results

### 3.1 Demographic and Perinatal Characteristics of the Index Cohort

In total, 1559 individuals were included, of whom 53% were female, totalling 824. The median age was 15 years, with a range of 4–52 years. Descriptive characteristics and perinatal parameters were summarised in Table 1 by the four study groups.

**TABLE 1:** Characteristics of the index cohort (*n* = 1559).

	Group 1	Group 2	Group 3	Group 4	*p*
	GA ≥ 37 weeks	GA 33–36 weeks	GA 29–3 weeks	GA ≤ 28 weeks	
Number of individuals	680	379	296	204	
Women	354 (52.1%)	202 (53.3%)	155 (52.4%)	113 (55.4%)	0.86
Age at examination (y), median [IQR]	16 [11.00, 27.00]	15 [10.00, 23.00]	16 [11.00, 24.00]	14 [9.00, 18.25]	< 0.001
Age 4–10 years, *n* (%)	160 (23.5%)	96 (25.3%)	62 (20.9%)	62 (30.4%)	< 0.001
Age 11–17 years, *n* (%)	220 (32.4%)	146 (38.5%)	118 (39.9%)	85 (41.7%)	< 0.001
Age 18–30 years, *n* (%)	174 (25.6%)	82 (21.6%)	76 (25.7%)	52 (25.5%)	< 0.001
Age 31–52 years, *n* (%)	126 (18.5%)	55 (14.5%)	40 (13.5%)	5 (2.5%)	< 0.001
Birth weight (*g*)	3407.21 ± 764.56	2238.11 ± 448.51	1535.61 ± 364.96	826.67 ± 240.82	< 0.001
Birth weight percentile	47.68 ± 35.03	30.83 ± 23.66	41.17 ± 24.31	37.62 ± 26.05	< 0.001
Severely SGA (BW percentile < 3)	70 (10.3%)	31 (8.2%)	9 (3.0%)	12 (5.9%)	< 0.001
Moderately SGA (BW percentile 3 to < 10)	70 (10.3%)	57 (15.0%)	19 (6.4%)	27 (13.2%)	< 0.001
AGA (BW percentile 10–90)	400 (58.8%)	285 (75.2%)	266 (89.9%)	163 (79.9%)	< 0.001
Moderately LGA (BW percentile > 90–97)	70 (10.3%)	3 (0.8%)	2 (0.7%)	2 (1.0%)	< 0.001
Severely LGA (BW percentile > 97)	70 (10.3%)	3 (0.8%)	0 (0.0%)	0 (0.0%)	< 0.001
Gestational age (weeks)	39.09 ± 1.39	34.52 ± 1.02	30.70 ± 1.11	25.89 ± 1.60	< 0.001
Preeclampsia (yes)	39 (5.7%)	45 (11.9%)	49 (16.6%)	34 (16.7%)	< 0.001
Placental insufficiency (yes)	15 (2.2%)	24 (6.3%)	9 (3.0%)	14 (6.9%)	0.001
Maternal smoking (yes)	30 (4.4%)	15 (4.0%)	18 (6.1%)	23 (11.3%)	0.001
HELLP syndrome (yes)	1 (0.1%)	14 (3.7%)	23 (7.8%)	11 (5.4%)	< 0.001
Gestational diabetes (yes)	51 (7.5%)	26 (6.9%)	23 (7.8%)	8 (3.9%)	0.32
Breastfeeding (yes)	494 (72.6%)	258 (68.1%)	183 (61.8%)	103 (50.5%)	< 0.001

Abbreviations: AGA, appropriate for gestational age; GA, gestational age; HELLP syndrome, haemolysis, elevated liver enzymes and low platelet count syndrome; LGA, large for gestational age; SGA, small for gestational age.

### 3.2 Maternal Birth Experiences by Gestational Age

There were significant differences across gestational age groups in various maternal experiences and outcomes related to childbirth (Table 2). Notably, the perception of the birth as an emergency was significantly more frequent in Group 4 (87.7%) compared to Group 1 (16.1%). The prevalence of psychological birth trauma was also higher in Group 4 (18.1%) compared to Group 1 (1.1%). Mothers in Group 4 reported the longest delay in cuddling their newborn (median 2.0 weeks). Preparation for discharge of their child was perceived as good by more mothers in Group 1 (90.4%) compared to Group 4 (82.1%).

**TABLE 2:** Characteristics of the maternal and paternal participants and their perception of the birth experience.

	Group 1	Group 2	Group 3	Group 4	*p*
	GA ≥ 37 weeks	GA 33–36 weeks	GA 29–32 weeks	GA ≤ 28 weeks	
*Mothers*	418	230	171	121	
Age at data collection (y)	50.1 ± 9.9	47.9 ± 9.2	49.0 ± 8.6	46.8 ± 9.2	0.003
Emergency birth (yes)	61/380 (16.1%^a^)	109/215 (50.7%)	119/153 (77.8%)	93/106 (87.7%)	< 0.001
Psychological birth trauma (yes)^b^	4/356 (1.1%)	11/182 (6.0%)	11/136 (8.1%)	17/94 (18.1%)	< 0.001
Cuddling after birth (weeks)	0.10 [0.10, 0.10]	1.00 [0.10, 1.00]	1.00 [1.00, 1.75]	2.00 [1.00, 3.00]	< 0.001
Good preparation for discharge (yes)	356/394 (90.4%)	170/212 (80.2%)	121/153 (79.1%)	87/106 (82.1%)	0.001
Frequency woken at night (in infant's first year of life)	2.06 ± 1.26	2.51 ± 1.42	2.22 ± 1.40	2.21 ± 1.25	0.001
Birth influence on childhood (yes)	57/372 (15.3%)	49/204 (24.0%)	57/148 (38.5%)	55/102 (53.9%)	< 0.001
Permanent impairment due to birth (yes)	17/386 (4.4%)	24/212 (11.3%)	34/148 (23.0%)	49/105 (46.7%)	< 0.001
Negative feelings towards motherhood (during birth or childhood)	16/380 (4.2%)	9/206 (4.4%)	12/141 (8.5%)	14/101 (13.9%)	0.001
*Fathers*	276	161	96	81	
Age at data collection (y)	51.9 ± 10.4	50.7 ± 9.0	50.2 ± 8.4	49.7 ± 9.8	0.21
Emergency birth (yes)	37/240 (15.4%)	69/139 (49.6%)	64/87 (73.6%)	65/73 (89.0%)	< 0.001
Psychological birth trauma (yes)^b^	1/191 (0.5%)	1/120 (0.8%)	0/79 (0.0%)	2/66 (3.0%)	0.21
Cuddling after birth (weeks)	0.10 [0.10, 0.10]	1.00 [0.10, 1.00]	1.00 [0.10, 1.00]	1.00 [1.00, 3.00]	< 0.001
Good preparation for discharge (yes)	216/231 (93.5%)	124/136 (91.2%)	75/84 (89.3%)	59/68 (86.8%)	0.31
Frequency woken at night (in infant's first year of life)	1.89 ± 1.76	2.09 ± 1.35	1.82 ± 1.14	1.99 ± 1.31	0.57
Birth influence on childhood (yes)	22/207 (10.7%)	19/122 (15.6%)	22/273 (27.2%)	27/75 (35.5%)	< 0.001
Permanent impairment due to birth (yes)	7/244 (2.9%)	11/136 (8.1%)	14/86 (16.3%)	27/72 (37.5%)	< 0.001
Negative feelings towards fatherhood (during birth or childhood)	3/61 (4.9%)	4/37 (10.8%)	0/12 (0%)	7/16 (43.8%)	< 0.001

Abbreviation: GA, gestational age.

^a^ Percentages are based on the number of valid responses, which vary slightly due to missing data.

^b^ Psychological birth trauma assessed through the Peri‐Trauma Inventory Questionnaire, which has a scale ranging from 0–52. A score of 23 was used as a cut‐off value for present birth trauma.

### 3.3 Paternal Birth Experiences by Gestational Age

Significant differences were also observed across gestational age groups in various paternal experiences (Table 2). The perception of the birth as an emergency was significantly more frequent in Group 4 (89.0%) compared to Group 1 (15.4%). Fathers in Group 4 reported the longest delay in cuddling their newborn (median 1.0 week), whereas fathers in Group 1 experienced the shortest delay (median 0.10 week). Preparation for discharge was perceived as good by a higher percentage of fathers in Group 1 (93.5%) compared to Group 4 (86.8%).

### 3.4 Differences Between Mothers and Fathers

Differences between mothers and fathers were evident in birth trauma, which was more common among mothers. Additionally, a higher percentage of fathers felt better prepared for hospital discharge of their children compared to mothers.

### 3.5 Time to First Cuddling After Birth (Over 50 Years)

**FIGURE 1:**
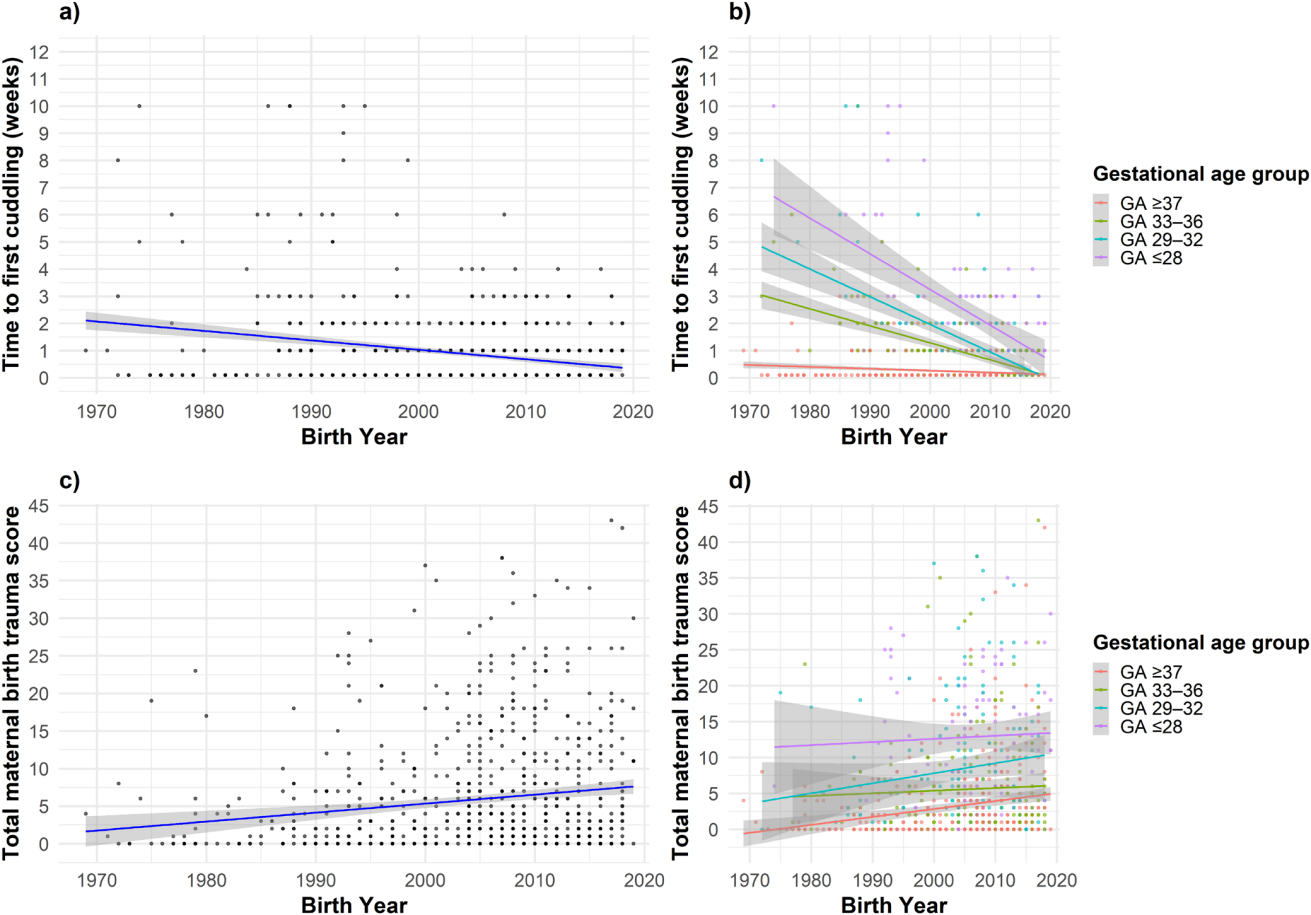
Weeks to first maternal cuddling over 50 years for the (a) total cohort, (b) stratified according to gestational age group and total maternal birth trauma stratified by (c) total cohort and (d) stratified according to gestational age group.

Figure 1a showed that the time to first cuddling reported by mothers decreased across decades. Trends were similar for mothers and fathers; however, in preterm groups, fathers reported earlier first cuddling (Figure S2). This difference should be interpreted with caution because the number of paternal responses was lower than the number of maternal responses.

Figure 1b showed a graded association between gestational age and time to first cuddling. Lower gestational age was associated with longer delays, although all preterm groups showed declines over the five decades.

### 3.6 Experiences of Childbirth and Perception of Motherhood

**FIGURE 2:**
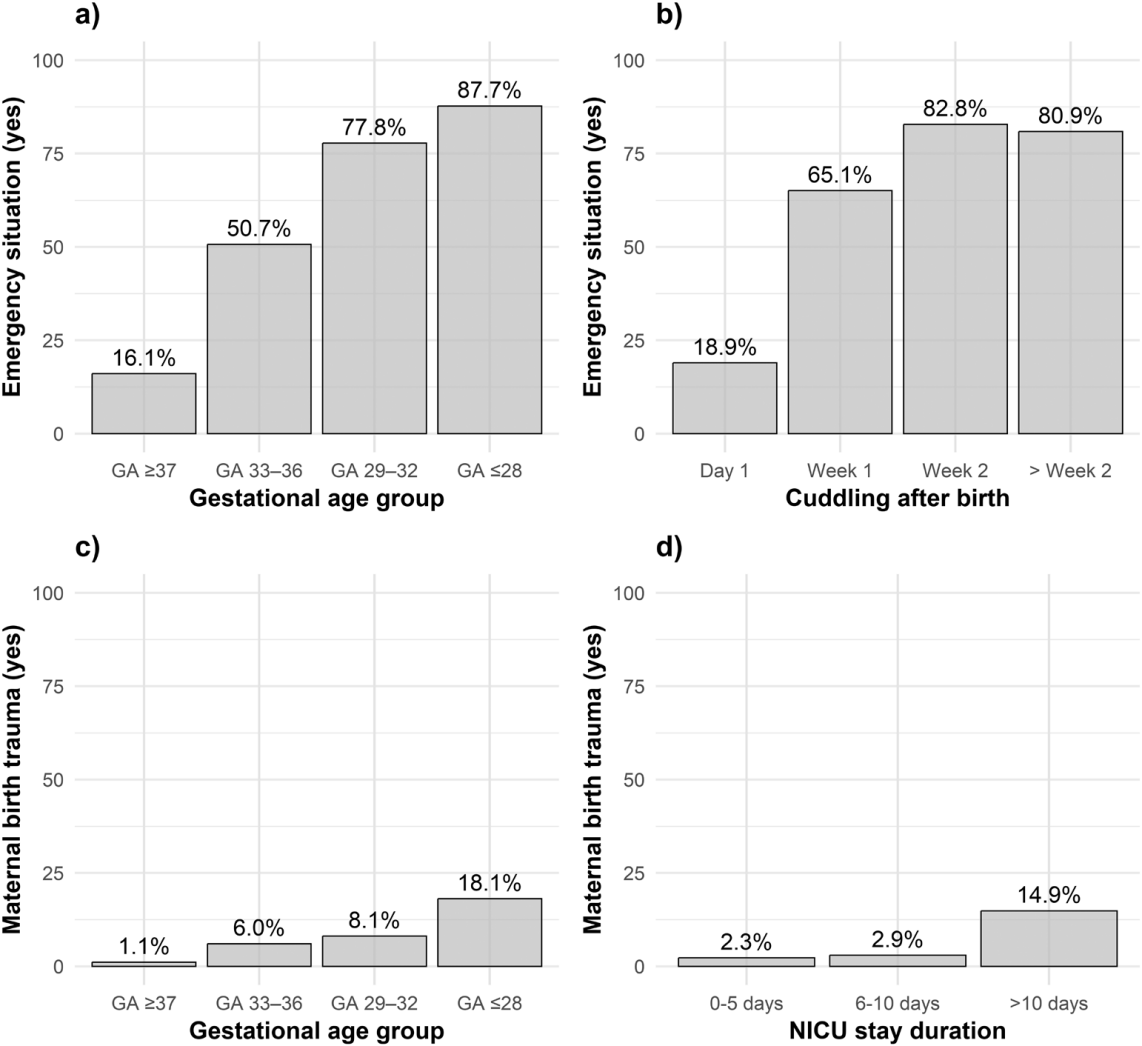
Maternal birth experiences stratified by various factors (unadjusted): (a) perception of birth as an emergency (yes) by gestational age, (b) perception of birth as an emergency (yes) by time to first cuddling after birth (in weeks), (c) experience of birth trauma by gestational age groups and (d) experience of birth trauma by length of neonatal intensive care unit stay (in days). ‘Week 1’ refers to Days 2–7 after birth, ‘Week 2’ to Days 8–14 and ‘> Week 2’ to Day 15 and later.

There was an overall increase in psychological maternal birth trauma scores over the years, with a noticeable upward trend (Figure 1c). Scores also rose within each gestational age group, except in the extremely preterm group, which showed consistently high values (Figure 1d). Psychological maternal birth trauma was significantly associated with individuals born with a gestational age ≤ 28 weeks and 29–32 weeks (Table 3). Logistic regression showed that each additional week of prematurity increased the risk of maternal birth trauma by 20%, whereas birth year had no significant effect once gestational age deficit was taken into account (see Table S1). Lower gestational age and delayed cuddling were correlated with lower preparedness for discharge, while a longer stay in neonatal intensive care was linked to greater preparedness. Multivariable logistic regression analysis further demonstrated that mothers with children born at a gestational age of ≤ 28 weeks, 29–32 weeks or 33–36 weeks had a significantly higher risk of experiencing the birth as an emergency situation compared to term births (Table 3, Figure 2). Delayed cuddling increased the likelihood of remembering the birth as an emergency, whereas a higher Apgar score was significantly associated with a reduction.

**TABLE 3:** Association analyses of mothers' birth experiences of infants born preterm and full‐term. Adjusted for the age of the mother and age of infant.

	Model 1 statistical predictors: GA		Model 2 statistical predictors: GA, BW percentile		Model 3 statistical predictors: GA, BW percentile, postnatal course	
	OR (95% CI)	*p*	OR (95% CI)	*p*	OR (95% CI)	*p*
Emergency birth						
GA ≤ 28 weeks	39.26 (21.22, 78.12)	< 0.001	37.86 (20.43, 75.41)	< 0.001	15.71 (4.78, 52.45)	< 0.001
GA 29–32 weeks	18.96 (11.85, 31.11)	< 0.001	18.77 (11.73, 30.80)	< 0.001	10.40 (5.72, 19.26)	< 0.001
GA 33–36 weeks	5.55 (3.77, 8.23)	< 0.001	5.23 (3.53, 7.83)	< 0.001	3.48 (2.27, 5.38)	< 0.001
BW percentile			1.00 (0.99, 1.00)	0.19	1.00 (0.99, 1.00)	0.62
Delayed cuddling after birth (weeks)					1.44 (1.16, 1.85)	0.002
Apgar score					0.77 (0.66, 0.90)	0.001
Duration of NICU (days)					1.00 (0.98, 1.01)	0.55
Psychological birth trauma						
GA ≤ 28 weeks	12.25 (4.16, 44.68)	< 0.001	11.94 (4.02, 43.87)	< 0.001	6.52 (1.21, 37.60)	0.03
GA 29–32 weeks	7.80 (2.61, 28.63)	< 0.001	7.75 (2.59, 28.45)	< 0.001	6.81 (2.00, 27.31)	0.003
GA 33–36 weeks	3.57 (1.10, 13.62)	0.04	3.47 (1.06, 13.35)	0.05	3.39 (1.01, 13.28)	0.06
BW percentile			1.00 (0.98, 1.01)	0.73	1.00 (0.98, 1.01)	0.64
Delayed cuddling after birth (weeks)					1.10 (0.85, 1.37)	0.42
Apgar score					1.24 (0.91, 1.73)	0.19
Duration of NICU stay (days)					1.01 (1.00, 1.03)	0.07
Good preparation for discharge						
GA ≤ 28 weeks	0.48 (0.27, 0.89)	0.02	0.51 (0.28, 0.95)	0.03	0.40 (0.12, 1.30)	0.13
GA 29–32 weeks	0.39 (0.23, 0.65)	< 0.001	0.40 (0.24, 0.66)	< 0.001	0.51 (0.27, 0.96)	0.03
GA 33–36 weeks	0.43 (0.26, 0.69)	< 0.001	0.46 (0.28, 0.75)	0.002	0.57 (0.34, 0.95)	0.03
BW percentile			1.01 (1.00, 1.01)	0.14	1.01 (1.00, 1.01)	0.16
Delayed cuddling after birth (weeks)					0.77 (0.66, 0.88)	< 0.001
Apgar score					1.14 (0.97, 1.33)	0.09
Duration of NICU (days)					1.02 (1.00, 1.03)	0.03

*Note:* A gestational age of ≥ 37 weeks was used as the reference category for the gestational age groups; psychological birth trauma was assessed through the Peri‐Trauma Inventory Questionnaire, which has a scale ranging from 0–52. A score of 23 was used as a cut‐off value; Models 1, Model 2 and Model 3 were adjusted for maternal age and child age.

Abbreviations: BW, birth weight; GA, gestational age.

**FIGURE 3:**
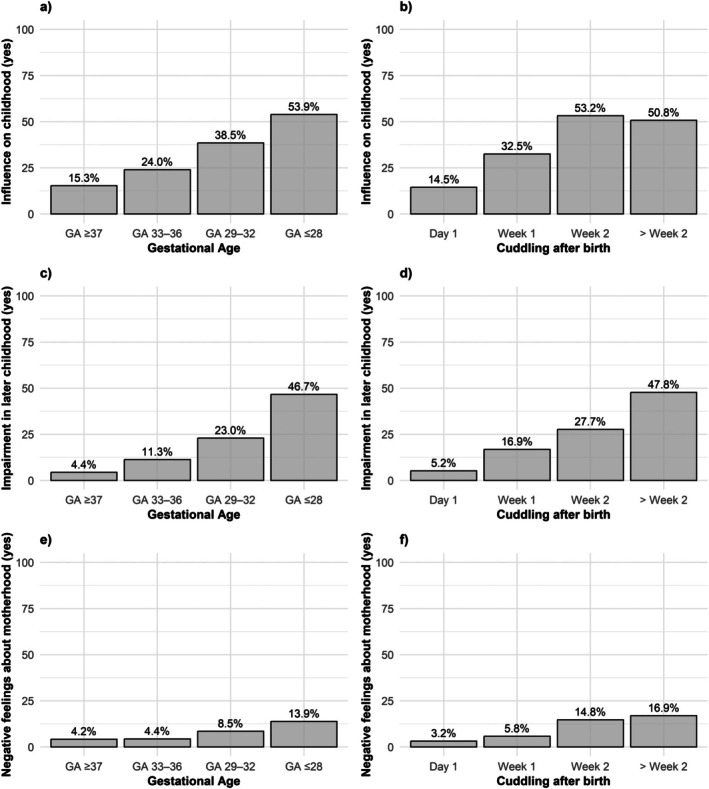
Impact of the birth situation on childhood and maternal perception of parenthood stratified by gestational age and cuddling after birth (unadjusted): (A) impact of the birth situation on childhood by gestational age, (B) impact of the birth situation on childhood by time to first cuddling after birth (in weeks), (C) impairment in childhood due to birth situation by gestational age, (D) impairment in childhood due to birth situation by time to first cuddling after birth (in weeks), (E) negative maternal feelings about parenthood by gestational age and (F) negative maternal feelings about parenthood by time to first cuddling after birth (in weeks). ‘Week 1’ refers to Days 2–7 after birth, ‘Week 2’ to Days 8–14 and ‘> Week 2’ to Day 15 and later.

Mothers in the preterm birth groups more often reported that the circumstances at birth had a lasting effect on the child's life during childhood (Table 4, Figure 3). They also indicated that permanent impairments due to birth conditions were more common in infants born extremely to late preterm. Longer neonatal intensive care stays were further associated with a higher likelihood of permanent impairments. Mothers of children born at 28 weeks or less more frequently described negative feelings towards motherhood, either at the time of birth or during the child's later childhood. In models that included both gestational age and time to first cuddling, only delayed cuddling remained associated with negative maternal experiences during this period.

**TABLE 4:** Association analyses of the perceived impact of birth experiences on the childhood and parenting of mothers of infants born preterm and full‐term. Adjusted for the age of the mother and age of infant.

	Model 1 statistical predictors: GA		Model 2 statistical predictors: GA, BW percentile		Model 3 statistical predictors: GA, BW percentile, postnatal course	
	OR (95% CI)	*p*	OR (95% CI)	*p*	OR (95% CI)	*p*
Impact of birth on later childhood (yes)						
GA ≤ 28 weeks	6.92 (4.26, 11.34)	< 0.001	6.45 (3.96, 10.61)	< 0.001	1.69 (0.61, 4.44)	0.30
GA 29–32 weeks	3.71 (2.39, 5.80)	< 0.001	3.66 (2.35, 5.72)	< 0.001	2.10 (1.20, 3.64)	0.01
GA 33–36 weeks	1.84 (1.19, 2.83)	0.01	1.64 (1.06, 2.55)	0.03	1.31 (0.82, 2.08)	0.26
BW percentile			0.99 (0.99, 1.00)	0.02	0.99 (0.99, 1.00)	0.04
Delayed cuddling after birth (weeks)					1.21 (1.05, 1.41)	0.01
Apgar score					0.95 (0.82, 1.10)	0.48
Duration of NICU (days)					1.01 (1.00, 1.02)	0.07
Permanent impairment due to birth						
GA ≤ 28 weeks	22.18 (11.90, 43.41)	< 0.001	21.83 (11.67, 42.86)	< 0.001	4.31 (1.45, 12.57)	0.01
GA 29–32 weeks	7.37 (3.97, 14.24)	< 0.001	7.39 (3.98, 14.28)	< 0.001	3.31 (1.60, 7.02)	0.001
GA 33–36 weeks	3.10 (1.61, 6.12)	< 0.001	3.04 (1.56, 6.05)	0.001	2.29 (1.16, 4.64)	0.02
BW percentile			1.00 (0.99, 1.01)	0.55	1.00 (0.99, 1.01)	0.83
Delayed cuddling after birth (weeks)					1.23 (1.06, 1.43)	0.007
Apgar score					0.91 (0.77, 1.08)	0.28
Duration of NICU stay (days)					1.01 (1.00, 1.03)	0.03
Negative feelings towards motherhood (during birth or childhood)						
GA ≤ 28 weeks	3.64 (1.68, 7.83)	< 0.001	3.37 (1.55, 7.29)	0.002	1.72 (0.38, 7.56)	0.47
GA 29–32 weeks	2.04 (0.89, 4.49)	0.08	2.01 (0.88, 4.42)	0.09	1.24 (0.44, 3.26)	0.67
GA 33–36 weeks	0.92 (0.36, 2.14)	0.85	0.81 (0.32, 1.90)	0.64	0.73 (0.28, 1.79)	0.50
BW percentile			0.99 (0.98, 1.00)	0.10	0.99 (0.98, 1.00)	0.17
Delayed cuddling after birth (weeks)					1.23 (1.03, 1.51)	0.05
Apgar score					1.01 (0.81, 1.31)	0.91
Duration of NICU (days)					1.00 (0.99, 1.02)	0.54

*Note:* A gestational age of ≥ 37 weeks was used as the reference category for the gestational age groups. Models 1, Model 2 and Model 3 were adjusted for maternal age and child age.

Abbreviations: BW, birth weight; GA, gestational age.

### 3.7 Comparisons of Mothers' and Fathers' Perceptions of Birth Experiences

There were differences in the mothers' and fathers' perceptions of birth experiences (Table S2). For both parents, a higher degree of prematurity and later first‐time cuddling were associated with a higher likelihood of experiencing birth as an emergency. Among mothers, a higher Apgar score decreased this likelihood for mothers, whereas among fathers, a longer stay in neonatal intensive care had the same effect.

In relation to preparation for discharge, mothers who cuddled later felt less prepared, while higher Apgar scores and longer neonatal intensive care stays increased their sense of preparedness. For both mothers and fathers, greater prematurity was associated with a stronger impression that the birth had affected childhood. Delayed cuddling strengthened this impression for mothers. Mothers also reported that permanent impairments were linked to greater prematurity and longer delays to cuddling, whereas fathers did not report such an association.

## 4 Discussion

This study explored how the degree of prematurity and birth‐related factors, such as duration of neonatal intensive care, Apgar scores and the timing of first cuddling, were related to parental perceptions of the birth experience. The timing of first cuddling over the past 50 years was also examined, with a particular focus on preterm infants. The study found that the time to first cuddling decreased across decades. Preterm birth increased the likelihood that mothers recalled the birth as an emergency and perceived it as having a lasting impact on childhood. Delayed cuddling was substantially associated with negative feelings towards motherhood, both at the time of birth and during later childhood.

### 4.1 Improvements in Early Cuddling Practices

There was a significant improvement in the time to first cuddling over the past 50 years. Several studies have shown that earlier skin‐to‐skin contact improves the health of premature babies and low birth weight babies [33, 34, 35]. For example, a study of preterm newborns with a birth weight of 2000 g or less reported that those who received Kangaroo Care for 24 h per day at the mother's chest had a lower, although not statistically significant, risk of death, fewer severe infections and shorter hospital stays than those who did not [33]. Another study involving infants with a birth weight of 1800 g or less found that an average of 16.9 h per day at their mother's chest was associated with lower mortality after 28 days compared with a control group who received an average of 1.5 h per day [34]. While the implementation of Kangaroo Care has not been fully adopted in Germany, our results indicate that the time until the first cuddle in preterm newborn groups has significantly decreased over the last 50 years. This trend is also reflected in local institutional developments. At our institution, the University Medical Center Mainz, a significant shift in practice occurred in the early 1990s. In 1993, a paradigm shift in neonatal care was initiated, driven primarily by the nursing leadership, particularly the head nurse and her team. Inspired by visits to pioneering units such as the Mautner Markhof Hospital in Vienna, staff began advocating for early physical contact between parents and their preterm infants. At that time, skin‐to‐skin contact with ventilated and clinically unstable neonates was still uncommon and required substantial improvisation and interprofessional coordination. The first such transfer involved a ventilated infant with oesophageal atresia who was placed on the mother's chest under close medical and nursing supervision. Although the medical team was concerned about the clinical risks, the transfer was successfully carried out in a tightly coordinated effort between nurses and physicians. This moment marked a fundamental cultural change in the unit, with skin‐to‐skin contact increasingly becoming part of standard care. The nursing team played a pivotal role in promoting these practices, and their close collaboration with medical staff enabled the gradual implementation of more family‐centred care.

This reflects a positive shift in care practices towards earlier skin‐to‐skin contact and this move towards earlier cuddling represents a step closer to these beneficial practices. A nationwide survey of German neonatal units with a response rate of 51% found that 98% of the units reported using some form of Kangaroo Care. Intermittent Kangaroo Care was more common than continuous Kangaroo Care, but both approaches contributed to improved parent–infant bonding and to greater stability in preterm infants [36].

### 4.2 Experiences at Childbirth

The mothers of individuals born preterm experienced psychological birth trauma more frequently than mothers of individuals born term. Eutrope et al. studied 100 mother–child pairs born at 32 weeks of gestation and found that 35% of the mothers had a high risk of trauma. This risk was correlated with the subjective experience of, and concern about, the infant's low birth weight [37]. Another study examined 50 mothers of very low birth weight infants, defined as less than 1500 g and born before 32 weeks of gestation, together with a control group of full‐term infants with normal birth weight. Mothers of very low birth weight infants scored higher for traumatic experience, anxiety and depressive symptoms at all four measurement points. These were 1–3 days, 14 days, 6 months and 14 months after birth compared with the control group [38]. In addition to the association between preterm birth and maternal psychological birth trauma, a longer stay in neonatal intensive care was also associated with trauma, consistent with previous findings [39, 40].

The present study revealed an increase in psychological maternal birth trauma over the last 50 years. However, this association was not directly attributable to the year of birth but rather was strongly associated with preterm birth. Improved medical care and the resulting higher survival rates of extremely preterm infants likely contribute to this trend [41]. In addition, greater awareness of mental health issues and the gradual destigmatisation of psychological difficulties may also be contributing factors [42].

Moreover, the mothers of individuals born preterm and those who experienced delayed first cuddling felt less prepared for hospital discharge. These associations may be explained by the increased stress and psychological trauma associated with delayed cuddling, which could disrupt the ideal development of maternal–infant attachment [12]. This was not the case for fathers, possibly due to the more intensive caregiving roles and expectations placed on mothers, leading to higher stress levels. Miles et al. [43] reported that mothers experienced significantly greater stress in the neonatal intensive care environment compared with fathers. This finding indicates that mothers of premature infants or those who experienced delayed cuddling need more specific support to feel adequately prepared for the time after discharge. Veronez et al. [44] underlined the importance of continuous support and education for mothers throughout hospitalisation and the discharge process to promote maternal autonomy and successful home adaptation. According to several studies, early cuddling improved the emotional well‐being of mothers and could have helped them to better prepare for caring for their child after discharge. Early cuddling was reported to promote interactions between mother and child and to reduce the trauma of separation. Kimkool et al. assessed mothers of extremely preterm babies (gestational age < 28 weeks) who were immediately separated from their mothers after birth and did not receive skin‐to‐skin contact in the neonatal intensive care unit. They found that cuddling facilitated early parent–infant interactions and reduced the trauma of separation [45]. Another study examined the effects of skin‐to‐skin contact on mother–infant interactions and maternal emotional stress. The authors found that greater skin‐to‐skin contact was linked to better interactions and lower maternal stress [46]. Early cuddling during hospitalisation is crucial for infant‐parent bonding and promoting the self‐confidence of mothers of preterm infants. However, the evidence supporting these benefits is largely based on studies investigating Kangaroo Care and may not directly apply to all forms of early contact.

Furthermore, the present study showed that mothers of individuals born preterm or mothers who cuddled later with their child felt that the birth had a long‐term impact on the child's childhood. This perception may have been shaped by the emotional challenges of delayed cuddling and by increased parental anxiety about the child's fragile health. Extended medical care, often required for preterm infants, could also have influenced development and the parent–child relationship over time [47].

### 4.3 Perception of Motherhood

This analysis highlights that early cuddling can strongly influence maternal perceptions of parenthood and delays in the first cuddle can negatively influence these views. This observation was consistent with previous research that demonstrated the importance of early physical contact for maternal mental health. Tessier et al. [48] showed that Kangaroo Care led to a different perception of mothers towards their child, with mothers in the Kangaroo Care group demonstrating greater responsiveness to their infants. These mothers also felt more competent in stressful situations compared with those in the traditional care group, which indicated greater resilience. Another study found that skin‐to‐skin contact immediately after birth yielded positive long‐term effects, including increased maternal sensitivity and enhanced self‐regulation in the child [49].

In summary, our findings suggest that early cuddling between mother and infant may play an important role in postnatal care. Delayed cuddling was associated with more negative maternal perceptions of motherhood and may influence emotional bonding with the infant as well as maternal confidence. Mothers who reported delayed first cuddling more often felt unprepared for discharge from the hospital. These associations point to the potential relevance of early physical closeness for supporting parental self‐confidence and reducing stress during the early stages of motherhood. Our study showed that the time until the first cuddle after birth has significantly decreased over the past 50 years for mothers and fathers, particularly in parents of preterm infants. This positive trend suggests that awareness of the importance of early skin‐to‐skin contact is growing, and corresponding measures are increasingly implemented. This development supports better preparation of mothers for their new role and fosters a healthier mother–child relationship from the very beginning.

## 5 Strengths and Limitations

This study had several limitations. Its single‐centre, hospital‐based design limited generalisability. Recruitment challenges and participant opt‐outs may have introduced selection bias. The predominance of white participants also restricted applicability to other ethnic groups. Reliance on retrospective parental recall of first cuddling introduced potential for memory‐related bias, especially given reports from up to 50 years ago. Although birth events are often vividly remembered, accuracy may have been affected by memory decay, reinterpretation or later parenting experiences. The wide age range of the cohort, from four to 52 years, added variation in memory reliability and birth context. While the extended study period enabled a diverse sample, it also spanned significant changes in neonatal care. Analyses were adjusted for maternal and child age to account for this. Still, recall bias, particularly among older participants, remained a limitation to consider. The use of binary response options, such as yes or no, likely reduced cognitive burden and recall distortion. Moreover, the variable ‘time to first cuddling’ was captured as a coarse estimate in weeks. While this approach allowed for historical comparisons across decades in preterm infants, we were unable to assess more precise information, such as the number of hours or minutes after birth or the duration of physical contact. This decision reflected the retrospective nature of the assessment, as exact recall of such detailed timing was unlikely to be reliable and could have increased response burden or recall bias. As such, we opted for broader but more feasible time categories. The Peritraumatic Distress Inventory, which was used in this study to assess parental distress related to the birth experience, was also applied retrospectively. Although the instrument was originally designed for the acute assessment of emotional responses shortly after a traumatic event, some studies have explored its retrospective use in the perinatal context, including shortly after childbirth [50, 51]. In the present study, the retrospective assessment extended over a considerably longer period, which had not been validated. Therefore, although the instrument captured relevant symptom dimensions, interpretation of results had to take into account the potential influence of recall bias and memory reinterpretation over time.

Finally, experiential items, such as the perceived long‐term influence of the birth or feelings towards early parenthood, were designed specifically for this study and have not been formally validated. These items were developed to capture subjective parental impressions and were therefore included as exploratory measures. Nonetheless, their nonvalidated status represented a limitation in terms of measurement precision and comparability with other research.

Despite these limitations, this study examined an extraordinarily large cohort of adults born preterm with varying degrees of prematurity alongside a substantial control group with descriptions provided by the parents. This multi‐informant design expanded on previous research. A thorough assessment of perinatal medical history was conducted through the review of medical charts, including data about first cuddling.

## 6 Conclusion

To our knowledge, this was the first study to demonstrate changes in the timing of first cuddling after birth across a five‐decade period, with a specific focus on preterm infants. The observed decline in time to first cuddling reflected growing clinical awareness and the increasing implementation of early skin‐to‐skin contact. Delayed cuddling was associated with more negative maternal perceptions of the birth experience and early motherhood. This finding highlights the importance of early physical contact for parental bonding and emotional adjustment. Promoting early cuddling, especially in the care of preterm infants, may help strengthen early parent–child relationships and support parental well‐being.

## Conflicts of Interest

Pfeiffer N receives financial support and grants from Novartis, Ivantis, Santen, Thea, Boehringer Ingelheim Deutschland GmbH & Co. KG, Alcon and Sanoculis. Schuster AK receives research support from Allergan, Bayer, Heidelberg Engineering, PlusOptix and Novartis. Fieß A, Hartmann A, Ernst M, Mildenberger E, Wackernagel D, Grabitz SD, Urschitz MS, Beutel, Gißler S, Tesarz J: none.

## Supporting information


**Table S1:** Association analyses of birth year and gestational age groups with psychological maternal birth.
**Table S2:** Association analyses of birth experiences of maternal and paternal participants with children born.
**Figure S1:** Flowchart of study inclusion and exclusion criteria.
**Figure S2:** Distribution of observed days to first cuddling. Panels a and b show histograms of days to first.

## Data Availability

The data that support the findings of this study are available on request from the corresponding author. The data are not publicly available due to privacy or ethical restrictions.
