# Supplementary material for: Timing of First Cuddling After Preterm Birth Improved Over Five Decades, While Delays Were Linked to Negative Maternal Experiences
Source: Acta Paediatr. 2025 Sep 27;115(1):202–16. doi: 10.1111/apa.70304 (PMC12687722; doi:10.1111/apa.70304)

List of content

Table S1. Association analyses of birth year and gestational age groups with psychological maternal birth trauma. Adjusted for age of mother.

Table S2. Association analyses of birth experiences of maternal and paternal participants with children born preterm and full-term. Adjusted for the age of mother, father and child.

Figure S1. Flowchart of study inclusion and exclusion criteria.

Figure S2. Distribution of observed days to first cuddling. Panels a) and b) show histograms of days to first cuddling reported by the mothers and fathers. Panels c) and d) display violin plots for the time to first cuddling by gestational age groups, reported by the mothers and fathers.

Table S1. Association analyses of birth year and gestational age groups with psychological maternal birth trauma. Adjusted for age of mother and age of child.

	Model 1<br>Statistical predictors: GA		Model 2<br>Statistical predictors: GA, BW percentile	
	OR<br>(95% CI)	p-value	OR<br>(95% CI)	p-value
**Psychological maternal birth trauma (yes)**				
Birth year	1.31<br>(0.81, 2.15)	0.28	1.32<br>(0.81, 2.18)	0.26
Gestational age deficit	1.20<br>(1.13, 1.28)	<0.001	1.20<br>(1.13, 1.28)	<0.001
BW percentile			1.00<br>(0.98, 1.01)	0.61
Legend: GA - gestational age; BW – birth weight				

Table S2. Association analyses of birth experiences of maternal and paternal participants with children born preterm and full-term. Adjusted for the age of mother, father and child.

	**Multivariable model**			
	Mother		Father	
	OR (CI_{95})	p	OR (CI_{95})	p
**Emergency situation birth**				
Gestational age deficit	1.31 (1.21, 1.43)	<0.001	1.36 (1.25, 1.50)	<0.001
BW percentile	1.00 (0.99, 1.01)	0.81	1.00 (0.99, 1.01)	0.83
Delayed cuddling after birth (weeks)	1.45 (1.07, 2.05)	0.03	1.50 (1.09, 2.12)	0.02
Apgar Score	0.78 (0.63, 0.96)	0.02	0.82 (0.65, 1.03)	0.09
NICU stay (days)	0.99 (0.97, 1.00)	0.12	0.98 (0.96, 0.99)	0.01
**Good preparation for discharge**				
Gestational age deficit	1.01 (0.92, 1.11)	0.81	0.90 (0.79, 1.03)	0.13
BW percentile	1.00 (0.99, 1.01)	0.46	1.00 (0.99, 1.01)	0.95
Delayed cuddling after birth (weeks)	0.63 (0.49, 0.78)	<0.001	0.85 (0.62, 1.24)	0.32
Apgar Score	1.27 (1.03, 1.57)	0.03	0.76 (0.49, 1.12)	0.19
NICU stay (days)	1.02 (1.00, 1.04)	0.03	1.01 (0.99, 1.04)	0.25
**Impact of birth situation on later childhood (yes)**				
Gestational age deficit	1.09 (1.01, 1.18)	0.03	1.12 (1.01, 1.24)	0.03
BW percentile	1.00 (0.99, 1.00)	0.21	1.00 (0.99, 1.01)	0.60
Delayed cuddling after birth (weeks)	1.22 (1.01, 1.52)	0.05	1.09 (0.86, 1.38)	0.48
Apgar Score	1.00 (0.82, 1.22)	0.97	1.15 (0.88, 1.54)	0.31
NICU stay (days)	1.01 (0.99, 1.02)	0.31	1.00 (0.99, 1.02)	0.73

Table S2. Association analyses of birth experiences of maternal and paternal participants with children born preterm and full-term. Adjusted for the age of mother, father and child.

**Permanent impairment due to birth**				
Gestational age deficit	1.14 (1.03, 1.26)	0.01	1.13 (0.99, 1.30)	0.06
BW percentile	0.99 (0.98, 1.00)	0.29	0.99 (0.97, 1.00)	0.11
Delayed cuddling after birth (weeks)	1.30 (1.06, 1.62)	0.01	1.17 (0.92, 1.50)	0.20
Apgar Score	1.01 (0.79, 1.31)	0.93	1.09 (0.79, 1.52)	0.62
NICU stay (days)	1.01 (1.00, 1.02)	0.22	1.02 (1.00, 1.04)	0.09

Legend: ICU stay – Intensive care unit stay; BW – birth weight; GA – gestational age

Birth trauma and negative feelings about parenthood were not compared due to a small number of cases in paternal participants.

Figure S1. Flowchart of study inclusion and exclusion criteria.

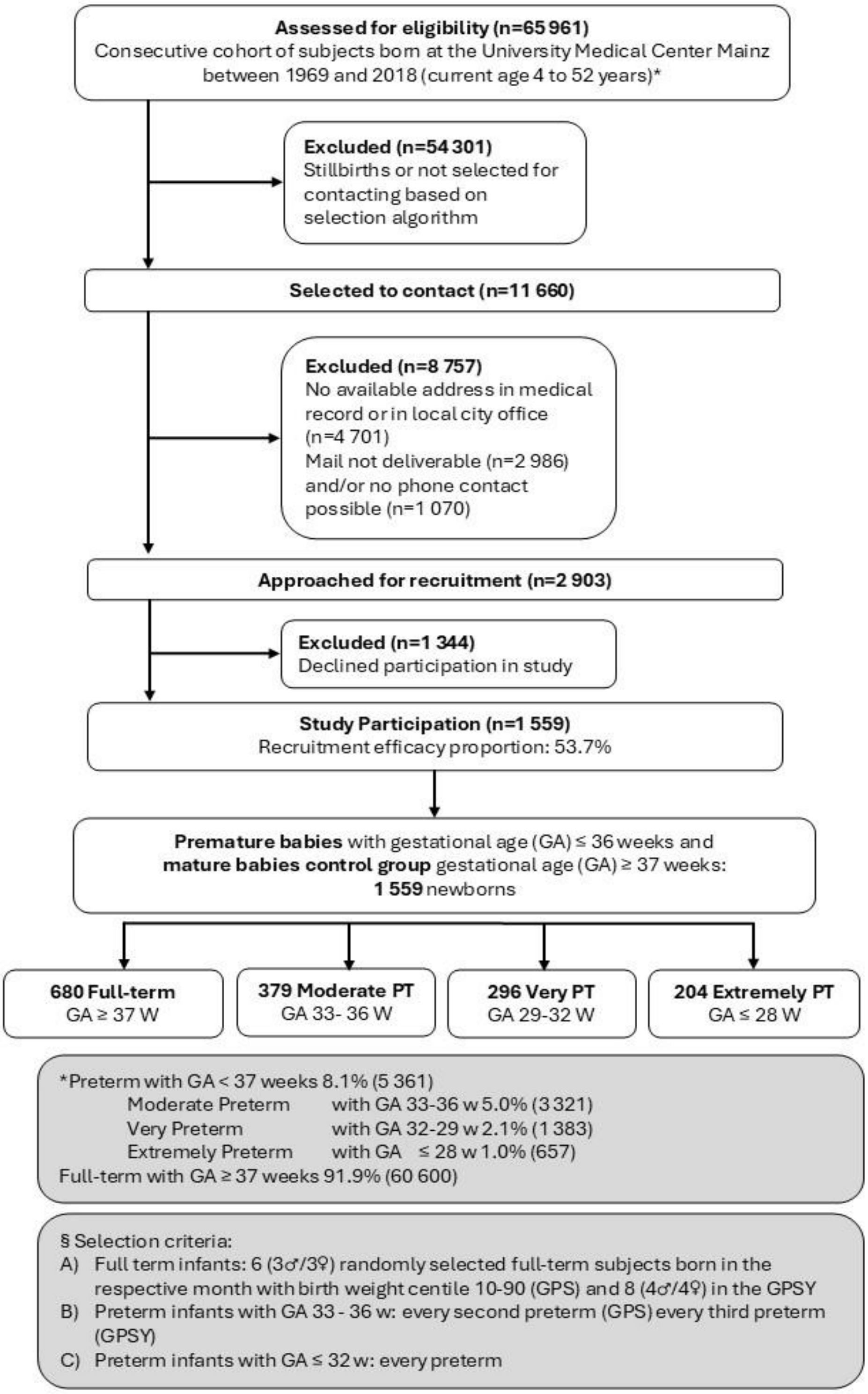

Figure S2. Distribution of observed days to first cuddling. Panels a) and b) show histograms of weeks to first cuddling reported by the mothers and fathers. Panels c) and d) display violin plots for the time to first cuddling by gestational age groups, reported by the mothers and fathers.

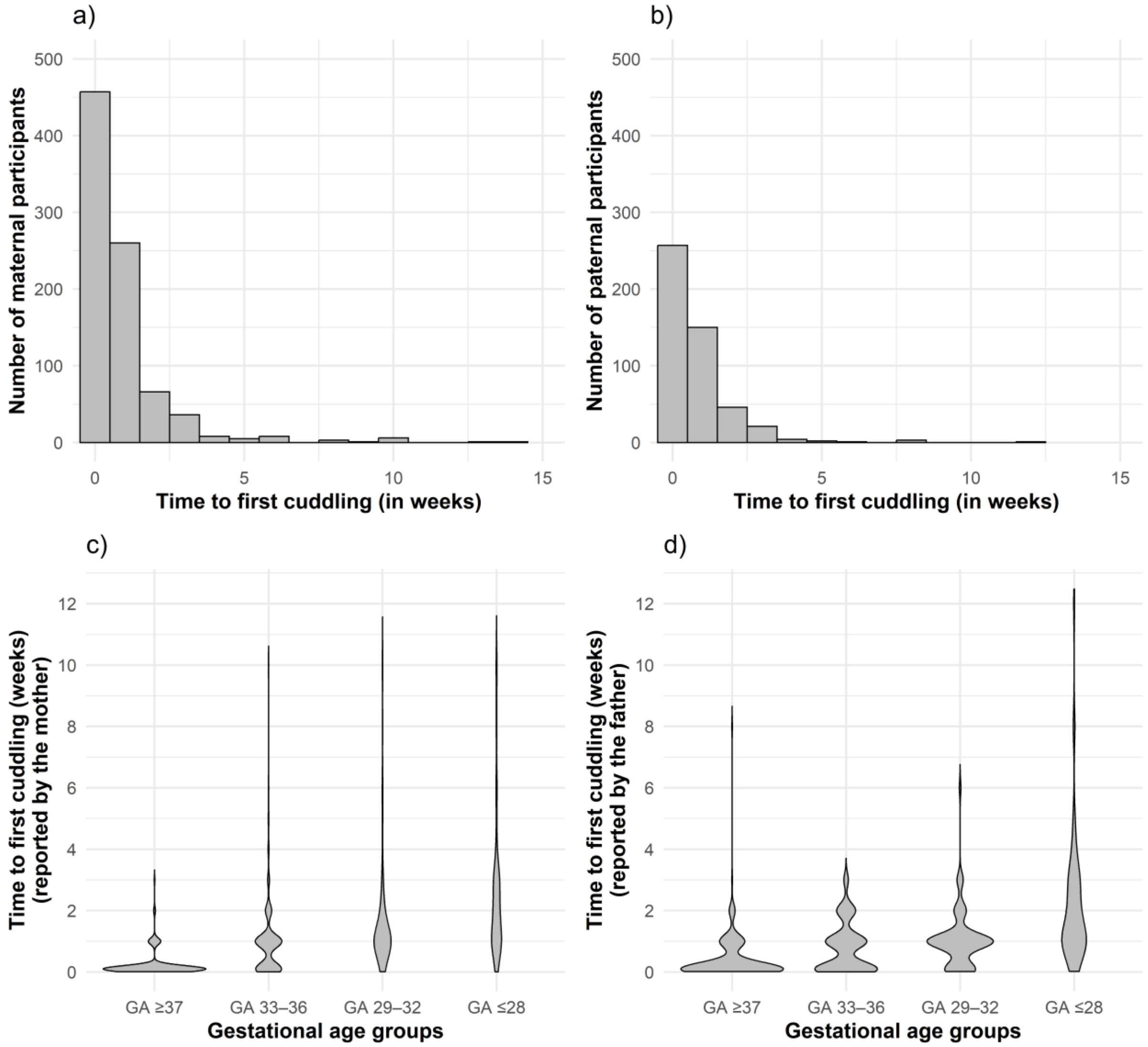

Supplement: Supplementary file 1 — Table S1: Association analyses of birth year and gestational age groups with psychological maternal birth. Table S2: Association analyses of birth experiences of maternal and paternal participants with children born. Figure S1: Flowchart of study inclusion and exclusion criteria. Figure S2: Distribution of observed days to first cuddling. Panels a and b show histograms of days to first. [file APA-115-202-s001.pdf]
